# A Physiological Chair

**Published:** 1859-03

**Authors:** 


### A PHYSIOLOGICAL CHAIR

Is made and sold by somebody—we know not who—pronounced by Prof. Fowler to be "the very thing" to make lazy young gentlemen and crooked-back girls sit erect; for, if they do not, they will slide off. We consider it the happiest thought of the times, put in practical shape, originating in the same broad platform of benevolence which led *George Comb* to devise how a natural shoe should be made. We never learned whether he was strikingly successful; but we think a New York "C," has, in the devise of a true physiological chair, accomplished a more important success than the great name just mentioned essayed to do when he gave his magnificent mind to the solution of the shoe problem. We advise all city mothers to call at once at our office, and see the chair.

